# Randomized phase 2 trial of intravenous oncolytic virus JX-594 combined with low-dose cyclophosphamide in patients with advanced soft-tissue sarcoma

**DOI:** 10.1186/s13045-022-01370-9

**Published:** 2022-10-21

**Authors:** Maud Toulmonde, Sophie Cousin, Michèle Kind, Jean-Philippe Guegan, Alban Bessede, Francois Le Loarer, Raul Perret, Coralie Cantarel, Carine Bellera, Antoine Italiano

**Affiliations:** 1 Early Phase Trials and Sarcoma Units, Department of Medical Oncology, Institut Bergonié, 229 Cours de l’Argonne, Bordeaux, France; 2 Department of Medical Imaging, Institut Bergonié, Bordeaux, France; 3 Explicyte, Bordeaux, France; 4 Department of Pathology, Institut Bergonié, Bordeaux, France; 5 Faculty of Medicine, University of Bordeaux, Bordeaux, France; 6 Inserm, Bordeaux Population Health Research Center, Epicene Team, UMR 1219, Univ. Bordeaux, 33000 Bordeaux, France; 7 Inserm CIC1401, Clinical and Epidemiological Research Unit, Comprehensive Cancer Center, Institut Bergonié, 33000 Bordeaux, France

**Keywords:** Soft-tissue sarcoma, Virus oncolytics, JX-594, Low-dose cyclophosphamide

## Abstract

JX-594 is an oncolytic vaccinia virus genetically modified to replicate selectively in tumor cells. Metronomic chemotherapy has shown preclinical synergy with oncolytic viruses. We report here the results of the METROMAJX which is a randomized phase II clinical trial investigating the combination of JX-594 combined with metronomic cyclophosphamide (arm 1) or metronomic cyclophosphamide (arm 2) in patients with advanced STS. A two-stage Simon design was used. JX-594 was administered intra-venously at the dose 1.109 every 2 weeks for the first 3 injections and then every 3 weeks. Cyclophosphamide was given orally at the dose of 50 mg BID 1 week on 1 week off. The primary endpoint was the 6-month non progression rate. 20 patients were included (arm 1:15, arm 2:5). The two most frequent toxicities were grade 1 fatigue and fever and grade 2 fatigue and grade 2 lymphopenia in arms 1 and 2, respectively. In arm 1, 12 patients were assessable for the efficacy analysis. None of them were progression-free at 6 months indicating that the first stage of the Simon's design was not satisfied. One patient out 4 assessable for efficacy was progression-free at 6 months in arm 2. High throughput analysis of sequential plasma samples revealed an upregulation of protein biomarkers reflecting immune induction such as CXCL10 and soluble CD8 antigen in arm 1. Systemic treatment with JX-594 is safe in patients with advanced STS. Further investigations are needed to improve immune response to oncolytic viruses and define their therapeutic potential in patients with STS.

**Supplementary Information:**

The online version contains supplementary material available at 10.1186/s13045-022-01370-9.

**To the Editor,**

Treatment of patients with advanced/metastatic soft-tissue sarcomas (STS) is dominated by conventional chemotherapy regimens with limited clinical activity. Therefore, there is an unmet need for combinations that would improve survival with limited toxicity.

Metronomic chemotherapy (MC) is defined as frequent and regular administration of low dose of a cytotoxic agent [1]. MC combines both antiangiogenic and immunomodulatory properties that make it a ‘niche’ multi-targeted therapy. STS represent one of the most frequent indications for routine use of metronomic cyclophosphamide in adult with solid tumors, with some clinical activity reported in prospective and retrospective studies [2].

JX-594 (Pexa-Vec; Jennerex Inc.) is a thymidine kinase (TK) gene-inactivated oncolytic vaccinia virus (OV) expressing granulocyte–macrophage colony stimulating factor (GM-CSF). It selectively destroys cancer cells through replication-dependent cell lysis and stimulation of antitumoral immunity. JX-954 also infects tumor-associated endothelial cells in a dose-dependent manner and induces vascular disruption. JX-594 has demonstrated a favorable safety profile and activity in preclinical and early clinical studies, both after intra-tumoral (IT), single and repeated intravenous (IV) injections at dose up to 3 × 10^9^ pfu/dose [3, 4].

TK gene expression is controlled by cellular E2F activity. Because of its selectivity for cells with up-regulation of TK gene expression, JX-594 could have a preferred activity in tumor cells with deregulation of pRB/E2F pathway, a deregulation that is frequent in STS [5].

Interestingly, metronomic cyclophosphamide has showed a synergistic effect on immuno-stimulation and promising clinical activity when combined with oncolytic adenovirus [6].

We therefore hypothesized that the association of metronomic cyclophosphamide and JX-594 could have a synergistic antitumor, and immuno-stimulating activity in patients with advanced STS.

Between April 2017 and February 2018, 20 patients with advanced STS were enrolled in the METROMAJX study, which is a randomized phase 2 trial (see Additional file 1 for further details). Eligible patients were randomly assigned (2:1) to receive either low dose cyclophosphamide (50 mg BID 1 week on 1 week off) or a regimen combining low dose cyclophosphamide 50 mg BID 1 week on, and JX-594 at the dose 1.109 every 2 weeks for the first 3 injections and then every 3 weeks. Fifteen patients were randomized in in the low dose cyclophosphamide and JX-594 combination arm (arm 1) and 5 in the low-dose cyclophosphamide arm (arm 2). Three patients were excluded from the efficacy analysis in arm 1 since they did not receive one complete cycle of treatment (Fig. 1). One patient was excluded from the efficacy analysis in arm 2 since the original diagnosis was not soft-tissue sarcoma but bone Ewing sarcoma (Fig. 1). Baseline patient characteristics are listed in Additional file 1: Table S1. Patients were heavily pre-treated: 10 patients (50% of the enrolled population) had received more than two previous lines, with a median number of previous lines of three (min:0–max:8) (Additional file 1: Table S1).

**Fig. 1:**
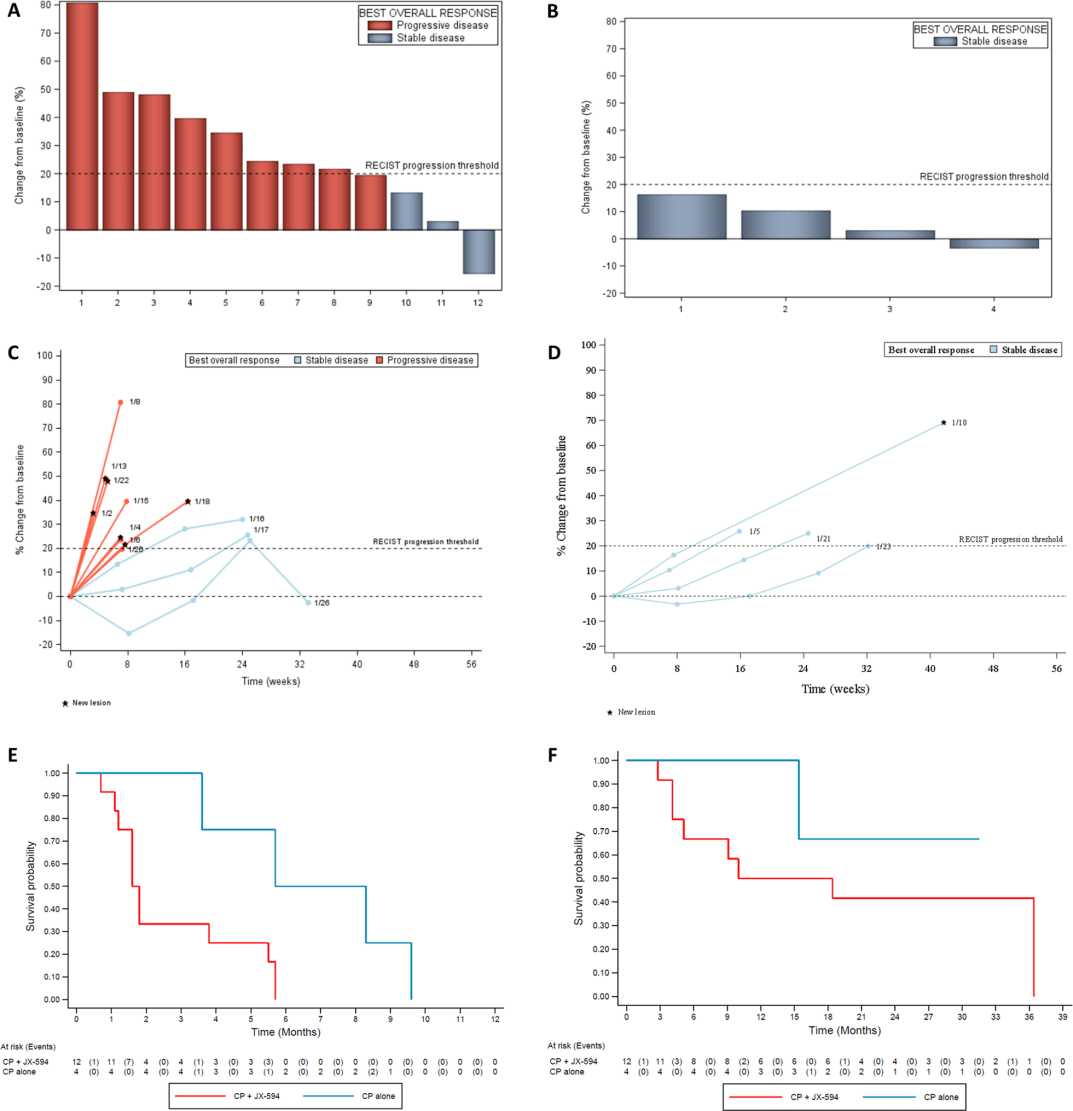
Efficacy of JX-594 combined with low-dose cyclophosphamide (**A**, **C**, **E**) and of low-dose cyclophosphamide (**B**, **D**, **F**) in patients advanced sarcomas. **A** and **B** Waterfall plot of the maximum change in tumor size in 12 and 4 patients with sarcomas and eligible for efficacy analysis in arms 1 (**A**) and 2 (**B**), respectively. **C** and **D** Representative spider plot illustrating changes from baseline in tumor burden according to RECIST criteria in patients from arm 1 (**C**) and arm 2 (**D**). **E** and **F** Kaplan–Meier curves associated with PFS (**E**) and OS (**F**) in arm 1 (red line) and arm 2 (blue line) (*n* = 16)

After a median follow-up of 31.5 months (95% CI 20.9–34.7, reverse Kaplan–Meier), all the patients discontinued treatment. In arm 1, discontinuation was related to disease progression in 14 cases and to patient’s decision in one case. In arm 2, discontinuation was related to disease progression in 4 cases and to patient’s decision in one case. In arm 1, 12 patients were assessable for the efficacy analysis (Additional file 1: Fig. S1). None of them were progression-free at 6 months indicating the first stage of the Simon's design was not satisfied. Best response as per RECIST criteria was stable disease for 3 patients and progressive disease for 9 patients (Fig. 1A–D). In arm 2, 4 patients were eligible for the efficacy analysis. Best response was stable disease for all of them (Fig. 1A–D).

8 patients died during the study, all of them were randomized in arm 1. Death was due to disease progression in all the cases. In arm 1, median progression-free survival and overall survival were 1.7 months (95% CI 1.1–5.5) and 14.2 months (95% CI 4.1–36.4) respectively (Fig. 1C, D). In arm 2, median progression-free survival was 7.0 months (95% CI 3.6–9.6) and not reached for overall survival respectively (Fig. 1E, F).

All the patients were included in the safety analysis. At the time of analysis, 47 cycles of JX-594 and metronomic CP had been administered, with a median of 2 cycles per patient (range 1–8) in arm 1. The most observed toxicities were grade 1 fever and grade 1 fatigue (Additional file 1: Table S2). Grade 3 toxicities were rare (observed in 2 patients) and included two cases of grade 3 fever and one case of grade 3 lymphopenia. No grade 4 toxicity was observed.

In arm 2, 43 cycles of metronomic CP were administered with a median of 10 cycles per patient (range 2–15). Grade 2 fatigue and grade 2 lymphopenia were the most frequent event adverse events (Additional file 1: Table S3). One patient experienced a grade 3 adverse event (anemia).

To evaluate the immune response to oncolytic virotherapy, we performed a proteomic analysis of plasma samples (see Additional file 1). Although only few changes in plasma proteome were observed between C1D8 and baseline (first administration of low dose cyclophosphamide), comparison of plasma samples between C1D22 and C1D8 (first injection of JX-594) revealed a significant upregulation of several proteins which reflect immune induction such as CD8A, and lymphocyte trafficking chemokines such as Cxcl10 but also of immunosuppressive cytokinges such as IL-18 and TGFbeta (Additional file 1: Fig. S2).

We report here the first phase II study investigating a virus genetically engineered for tumor-selective replication administered systemically in patients with STS. Although we observed low clinical activity, the results of our study pave the way for innovative approach to be evaluated in patients with advanced STS.

By analyzing transcriptomic data from > 600 soft-tissue sarcomas (STSs), we found that up to 60% of STS are poorly infiltrated by immune cells and have low expression of PD-L1 on their surface [7]. By activating an immune response to the tumor cells due to viral infection, OVs have the potential to turn the “cold” tumor microenvironment to “hot” and to sensitize tumors to immune checkpoint inhibitors as recently demonstrated in a pre-clinical model of triple negative breast cancer [8]. Interestingly, by using plasma proteomics, we have found that JX-594 treatment was associated with an upregulation of cytokines involved in anti-tumor immune response such as CXCL10, a chemokine playing a crucial role in recruiting activated T cells into sites of tissue inflammation and with the presence of CD8+ [9]. Interestingly, we found an increase in soluble CD8 antigen (CD8A) which is released in response to lymphocyte activation. Overall, these findings agree with those of a recent study investigating the immunomodulatory effect of JX-594 given before surgical resection of metastases in patients with colorectal cancer and melanoma. However, we also found an upregulation of immunosuppressive cytokines such as TGFb and IL18 revealing the potential complexity of the immunomodulatory effects of JX-594 and their impact of tumor microenvironment.

A recent study has reported the results of a single-center, phase II which investigated the combination of talimogene laherparepvec (T-VEC), an oncolytic immunotherapy derived from a modified human herpes simplex virus type 1, with pembrolizumab in patients with advanced STS [10]. T-VEC was administered intra-tumorally. 20 patients were enrolled in the trial. Most of them had locally advanced disease. Objective responses were observed in 30% of patients across 5 different histological subtypes. Given these promising results, whether the combination of JX-594 with an immune checkpoint inhibitor could be associated with meaningful clinical activity is therefore worth to investigate. A clinical trial assessing the combination of intra-tumoral JX-594 with the PD-L1 antagonist avelumab in patients with STS is ongoing (NCT02630368).

Although the number of patients enrolled in arm 2 was limited, due to fact that the accrual stopped at the end of the first stage, our data confirmed the results from retrospective studies suggesting that metronomic chemotherapy may have some clinical activity in patients with advanced sarcoma [2]. Indeed, all the patients enrolled in our study had disease progression confirmed at inclusion based on central review of two imaging performed at less than a 6-month interval. Interestingly, all of them had stable disease as best response and one of them was progression free at 6 months. Our study is the first one assessing prospectively the efficacy of metronomic cyclophosphamide in patients with advanced STS. Based on these preliminary results, a phase 3 study is currently comparing the efficacy of doxorubicin versus metronomic cyclophosphamide in patients aged more than 65 years old with metastatic STS (NCT04757337).

In conclusion, our study confirms that systemic administration of a genetically modified oncolytic virus is safe in patients with advanced STS. The role of the combination with other immune-oncology agents such as immune checkpoint inhibitors and the identification of the population of patients who are more likely to benefit from this approach are questions of major interest.

## Supplementary Information

**Additional file 1**. Supplementary methods.

## Data Availability

The datasets generated during and/or analyzed during the current study are not publicly available due to the clinical and confidential nature of the material but can be made available from the corresponding author on reasonable request.

## Abbreviations

CP: Circulating tumor cell
PFS: Progression-free survival
STS: Soft-tissue sarcoma
TK: Thymidine kinase
OS: Overall survival
