# Supplementary material for: Randomized phase 2 trial of intravenous oncolytic virus JX-594 combined with low-dose cyclophosphamide in patients with advanced soft-tissue sarcoma
Source: J Hematol Oncol. 2022 Oct 21;15:149. doi: 10.1186/s13045-022-01370-9 (PMC9585864; doi:10.1186/s13045-022-01370-9)
Supplement: Supplementary file 1 — Additional file 1. Supplementary methods. [file 13045_2022_1370_MOESM1_ESM.docx]

**PATIENTS AND METHODS**

METROMAJX is a non-comparative, randomized, open-label, phase 2 trial. Adults with metastatic or unresectable locally advanced, histologically confirmed soft-tissue sarcomas, measurable disease as per RECIST criteria [32] and laboratory tests of adequate organ function were eligible for this study. All the patients had to have documented disease progression confirmed after central review by two imaging obtained at less than 6 months interval. Eligible patients were randomly assigned (2:1) to receive either low dose cyclophosphamide (50 mg BID one week on one week off) or a regimen combining low dose cyclophosphamide 50 mg BID one week on, and JX-594 at the dose 1.10^9^ every 2 weeks for the first 3 injections and then every 3 weeks, with a web-based randomization system (TenAléa software). Once the randomization was completed, the investigator received an automated email confirmation with the group of treatment allocated. Randomization was stratified according to investigational center and number of metastatic sites. A minimization randomization method was used to avoid substantial imbalance between the groups. Patients and investigators were not masked to treatment allocation. Patients received the allocated treatment until disease progression, unacceptable toxicity, death, or physician or patient decision. Tumor lesions were assessed according to RECIST v1.1 at baseline within 21 days before treatment onset and then every 6 weeks until disease progression or start of another treatment. All response had to be confirmed by repeating imaging > 4 weeks. Safety was monitored by assessing all adverse events (AE) continuously through the study. This study was approved by the institutional ethics committee of Institut Bergonié (Comité de Protection des Personnes Sud-Ouest et Outre Mer III). All patients provided written informed consent before enrollment in the study. The corresponding author had full access to the data and had final responsibility for the decision to submit the manuscript for publication. Clinical Trial registration: NCT02630368

**Plasma proteomics**

Proteomic profiling of plasma samples collected at baseline (just before first administration of low dose cyclphophamide), C1D8 (first injection of JX-594) and C1D22 was assessed using the Olink Target 96 Inflammation panel (Olink Proteomics AB, Uppsala, Sweden) according to the manufacturer's instructions. The resulting DNA sequence was subsequently detected and quantified using a microfluidic real-time PCR instrument (Biomark HD, Fluidigm). Data were quality controlled and normalized using an internal extension control and an inter-plate control, to adjust for intra- and inter-run variation. The final assay read-out is presented in Normalized Protein eXpression (NPX) values, which is an arbitrary unit on a log2-scale where a high value corresponds to a higher protein expression. All assay validation data (detection limits, intra- and inter-assay precision data, etc) are available on manufacturer's website (www.olink.com). Differences in plasma collected at baseline, C1D8 and C1D22 were tested using a limma paired t-test (R package v3.48.3) and proteins with a fold change >1.25 and an adjusted p value (BH) <0.05 were extracted. Venn diagram was drawn using the ggvenn R package (v0.1.9)

**Statistical consideration**

The primary endpoint was the 6-month non progression rate defined as the percentage of patients remaining alive and progression-free at 6 months after treatment onset as per RECIST 1.1[32] and based on centrally reviewed radiological data by an independent expert radiologist blinded to the treatment.

Two patients were randomized in the experimental arm (cyclophosphamide with JX-594) for one patient randomized in the standard arm (cyclophosphamide alone).

For the experimental arm, a two-stage optimal Simon’s design was used, [33] with 43 eligible patients needed to distinguish a favorable true 6-month non progression rate of 40% (H1) from a null rate of 20% (H0) with 80% power, and 5% one-sided type I error rate in the combination arm. Following the inclusion of the first 13 assessable patients, if ≤ 3 patients were progression-free [complete response (CR), partial response (PR), or stable disease (SD)] at 6 months, the study would be terminated early. Otherwise, the second group of 30 subjects would be recruited. If at the end of recruitment, ≥ 13 patients of the 43 assessable patients were progression-free at 6 months, combination of low-dose cyclophosphamide with JX-594 would be considered worth further testing in STS. Given the 2:1 randomization ratio, 22 patients were planned to be included in the low-dose cyclophosphamide, with no formal statistical test for this arm. Secondary endpoints included safety by CTCAE v4.0, objective response defined as CR or PR as per RECIST 1.1, 1-year progression-free survival (PFS), and 1-year overall survival (OS). PFS was defined from start of treatment to time of progression or death (from any cause). OS was defined from start of treatment to death (from any cause) or last patient contact. Patients alive and progression-free were censored at the date of last follow-up. All enrolled patients who received at least one dose of one of cyclophosphamide or JX-594 were eligible for safety analyses. To be assessable for the primary efficacy endpoint, a subject had to meet eligibility criteria and had received at least one complete or two incomplete treatment cycles.

**ADDITIONAL FILE TABLES**

| **Additional file1: Table S1. Patient characteristics** | | |
| --- | --- | --- |
|  | **Arm 1 (n=15)** | **Arm 2 (n=5)** |
| **Variable** |  |  |
| **Gender, n (%)** |  |  |
| Male | 5 (33.3) | 3 (60.0) |
| Female | 10 (66.7) | 2 (40.0) |
| **Age** |  |  |
| Median, years (range) | 62 (36–78) | 65 (44–82) |
| **ECOG PS, n (%)** |  |  |
| 0 | 6 (40.0) | 4 (80.0) |
| 1 | 9 (60.0) | 1 (20.0) |
| **Histological subtype (%)​** |  |  |
| Well differentiated/dedifferentiated liposarcomas | 3 (20.0)​ | 0 (0.0) |
| Leiomyosarcomas | 7 (46.7) | 2 (40.0) |
| Other histotypes* | 5 (33.3) | 3 (60.0) |
| **Grade (%)** |  |  |
| 2 | 6 (40.0) | 0 (0.0) |
| 3 | 4 (26.7) | 1 (20.0) |
| Unknow | 5 (33.3) | 4 (80.0) |
| **Stage, n (%)** |  |  |
| Locally advanced | 2 (13.3) | 1 (20.0) |
| Metastatic | 13 (86.7) | 4 (80.0) |
| **Prior lines of chemotherapy n (%)​** |  |  |
| 0 | 3 (20.0) | 0 |
| 1​ | 1 (6.7) | 0 |
| 2​ | 4 (26.7) | 2 (40.0) |
| > 2​ | 7 (46.6) | 3 (60.0) |

***Other histotypes arm 1:** endometrial stroma tumor (n=1), epithelioid sarcoma (n=1), solitary fibrous tumor (n=1), undifferentiated pleomorphic sarcoma (n=2); **arm 2**: Ewing sarcoma (bone), solitary fibrous tumor (n=1), undifferentiated pleomorphic sarcoma (n=1). PS= performance status

**Additional file 1: Table S2: Number of patients presenting at least one adverse effect related to Cyclophosphamide and/or JX-594 by AE term, SOC and grade in Arm 1 (n=15)**

| **Maximum intensity** | | | | | | |
| --- | --- | --- | --- | --- | --- | --- |
|  | **Grade 1** | | **Grade 2** | | **Grade 3** | |
|  | **N** | **%** | **N** | **%** | **N** | **%** |
| Anemia | 2 | 14.3 | 5 | 35.7 | . | . |
| Diarrhea | 1 | 7.1 | . | . | . | . |
| Dry mouth | 1 | 7.1 | . | . | . | . |
| Mucositis oral | 1 | 7.1 | 1 | 7.1 | . | . |
| Nausea | 4 | 28.6 | . | . | . | . |
| Vomiting | 2 | 14.3 | . | . | . | . |
| Chills | 1 | 7.1 | . | . | . | . |
| Fatigue | 9 | 64.3 | 4 | 28.6 | . | . |
| Fever | 10 | 71.4 | 8 | 57.1 | 2 | 14.3 |
| Papulopustular rash | 6 | 42.9 | . | . | . | . |
| Other infections and infestations | 1 | 7.1 | . | . | . | . |
| Alanine aminotransferase increased | 1 | 7.1 | . | . | . | . |
| Lymphopenia | 3 | 21.4 | 1 | 7.1 | 1 | 7.1 |
| Anorexia | . | . | 1 | 7.1 | . | . |
| Dysgeusia | 1 | 7.1 | . | . | . | . |
| Cough | 1 | 7.1 | . | . | . | . |
| Dyspnea | 1 | 7.1 | . | . | . | . |

| Dry skin | 2 | 14.2 | . | . | . | . |
| --- | --- | --- | --- | --- | --- | --- |

**Additional file 1: Table S3: Number of patients presenting at least one adverse effect related to Cyclophosphamide and/or JX-594 by AE term, SOC and grade in Arm 2 (n=5)**

| **Maximum intensity** | | | | | | |
| --- | --- | --- | --- | --- | --- | --- |
| **Grade 1** | | | **Grade 2** | | **Grade 3** | |
| **N** |  | **%** | **N** | **%** | **N** | **%** |
| Anemia | 2 | 40.0 | . | . | 1 | 20.0 |
| Vertigo | 1 | 20.0 | . | . | . | . |
| Diarrhea | 2 | 40.0 | . | . | . | . |
| Mucositis oral | 1 | 20.0 | 1 | 20.0 | . | . |
| Nausea | 1 | 20.0 | . | . | . | . |
| Fatigue | 2 | 40.0 | 3 | 60.0 | . | . |
| Fever | 2 | 40.0 | 2 | 40.0 | . | . |
| Malaise | 1 | 20.0 | . | . | . | . |
| Rash pustular | 1 | 20.0 | . | . | . | . |
| Alanine aminotransferase increased | 1 | 20.0 | . | . | . | . |
| Aspartate aminotransferase increased | 1 | 20.0 | . | . | . | . |
| Lymphocyte count decreased | 1 | 20.0 | 3 | 60.0 | . | . |
| Platelet count decreased | 1 | 20.0 | . | . | . | . |
| Other musculoskeletal and connective tissue disorder | 1 | 20.0 | . | . | . | . |
| Dysgeusia | . | . | 1 | 20.0 | . | . |
| Dry skin | 1 | 20.0 | . | . | . | . |

**ADDITIONAL FILE FIGURES**

**Additional file 1: Figure S1.** Flow chart of the study. Three patients were not assessable for efficacy in Arm 1. Patient 1-3, 1-9 and 1-24 did not receive one complete cycle of treatment. One patient in arm 2 (patient 1-14) was not eligible (diagnosis of bone sarcoma). CP= cyclophosphamide


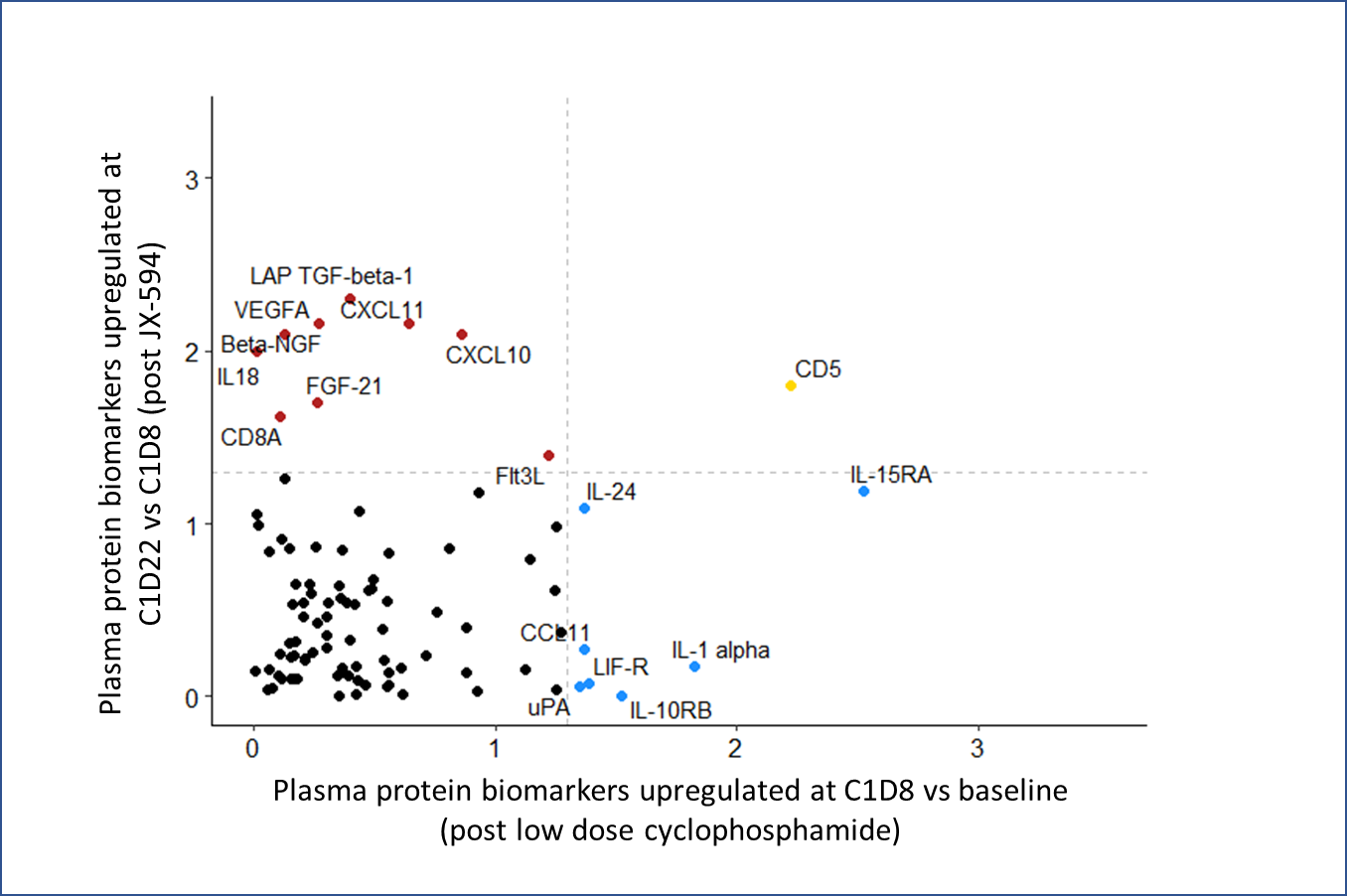


**Additional file 1: Figure S2**. Plasma protein biomarkers upregulated after administration of low-dose cyclophosphamide (x axis) or JX-594 (y axis) in patients enrolled in arm 1.
